# A Full Diagnostic Process for the Orthodontic Treatment Strategy: A Documented Case Report

**DOI:** 10.3390/dj8020041

**Published:** 2020-05-06

**Authors:** Antonino Lo Giudice, Lorenzo Rustico, Vincenzo Ronsivalle, Paola Spinuzza, Alessandro Polizzi, Angela Mirea Bellocchio, Simone Scapellato, Marco Portelli, Riccardo Nucera

**Affiliations:** 1 Department of Medical-Surgical Specialties—Section of Orthodontics, School of Dentistry, University of Catania, Policlinico Universitario “V. Emanuele,” Via Santa Sofia 78, 95123 Catania, Italy; nino.logiudice@gmail.com (A.L.G.); vincenzoronsivalle@hotmail.it (V.R.); alexpoli345@gmail.com (A.P.); simonescapellato@hotmail.com (S.S.); 2 Department of Biomedical and Dental Sciences and Morphofunctional Imaging, Section of Orthodontics, School of Dentistry, University of Messina, Policlinico Universitario “G. Martino,” Via Consolare Valeria, 98123 Messina, Italy; paolaspinuzza@gmail.com (P.S.); angelamirea@live.it (A.M.B.); mportelli@unime.it (M.P.); riccardo.nucera@gmail.com (R.N.)

**Keywords:** orthodontic treatment, self-ligating appliances, extraction, periodontal health, relapse

## Abstract

The need for extractions in orthodontic treatment has always been a controversial topic. However, to date there is not a specific clinical guideline that can help the clinicians deciding to plan an extractive or a non-extractive orthodontic treatment. In this respect, clinicians must deal with patients’ occlusal, functional, periodontal and aesthetics characteristics before planning an orthodontic treatment including extraction. Considering the absence of specific guidelines, the choice to extract teeth or not is complicated, particularly in borderline cases. In this case report, we present a borderline case of a patient with the skeletal Class III pattern and significant crowding in both arches that could be treated with or without extraction, illustrating the diagnostic and decision-making processes that were conducted for the orthodontic treatment strategy.

## 1. Introduction

The need for extractions in orthodontic treatments has been a controversial subject from the beginning of orthodontics as a medical specialty.

At the beginning of the 20th century, Angle believed that all 32 teeth could be accommodated in the jaws and that bone would form around the teeth in their new position, refusing any need of extractions [1]. In the mid-20th century, Begg, in Australia, studying aboriginal skulls, noted a large amount of occlusal and more importantly interproximal wear. He suggested that normal occlusion can develop only when the amount of tooth substance, relative to jaw size, is so small that attrition is not required to reduce the tooth substance, implying the need for premolar extractions [2]. At the same time, Tweed, in the USA, disappointed from the results he achieved with a non-extraction orthodontic treatment, decided to orthodontically retreat patients who had experienced relapse by the extraction of four premolars [3]. His outcomes resulted in a change of orthodontic philosophy to extraction-based techniques. Later, in the second half of the 20th century, the extraction debate was raised again, Tweed’s extraction philosophy was stigmatized for his aesthetics outcomes, and in contrast with it, there was the spread of Cetlin’s non-extraction philosophy [4].

Clinicians must deal with patients’ occlusal, functional, periodontal and aesthetics characteristics before planning an orthodontic treatment including extraction [5]. Nowadays, comprehensive clinical guidelines suggesting when orthodontic treatment plans should include teeth extraction are missing, thus there is a lack of a specific decision algorithm that can guide the orthodontist towards the correct treatment choice of whether to extract or not [6].

Extractions can be planned to reach several treatment objectives: the alleviation of crowding, the reduction in the dentoalveolar protrusion of anterior teeth and the overlying soft tissue, to improve lip competence, and the dental compensation of skeletal disharmonies [7]. Extractions do not negatively affect the final occlusion, post-treatment stability, soft tissue profile, smile aesthetics or facial growth pattern, especially when performed after an adequate diagnostic evaluation [8,9,10].

In the extraction vs. non-extraction decision-making process, clinicians must take into account not only the relative benefits but also the possible side-effects related to both approaches. In this respect, treatment including extractions, if improperly planned, may negatively affect the soft tissue profile (in particular, labial projection) and reduce the inter-premolar width, avoiding a complete filling of the buccal corridors [11]. Conversely, when the dentoalveolar discrepancy is treated without extraction, potentially negative side-effects may be excessive teeth flaring that according to the pretreatment periodontal health and to the biomechanics used can determine the alterations to the periodontal tissues, becoming a risk factor for the loss of the vestibular bone and the formation of gingival recessions [12,13].

Considering the lack of comprehensive guidelines, the choice to extract teeth or not is complicated, particularly in borderline cases, i.e., those clinical situations which recognize a very precise diagnosis but expose the clinician to very contrasting and sometimes divergent therapeutic solutions [14].

In this respect, in this article, we present a borderline case of a patient with the skeletal Class III pattern and significant crowding in both arches that could be treated with or without extraction to illustrate the diagnostic process that was conducted during the decision-making process for the orthodontic treatment strategy.

## 2. Case Report

### 2.1. Diagnosis and Etiology

**Figure 1:**
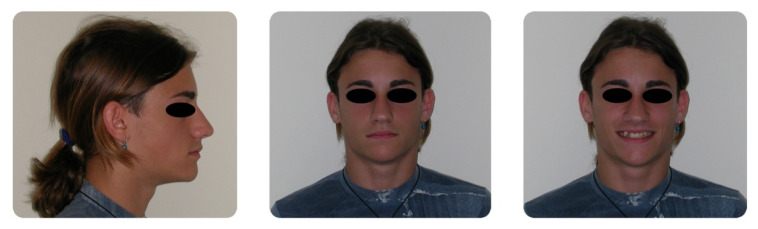
Pretreatment extraoral photographs.

**Figure 2:**
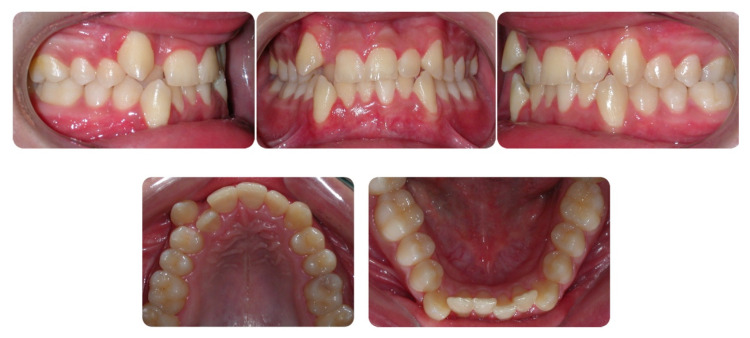
Pretreatment intraoral photographs.

**Figure 3:**
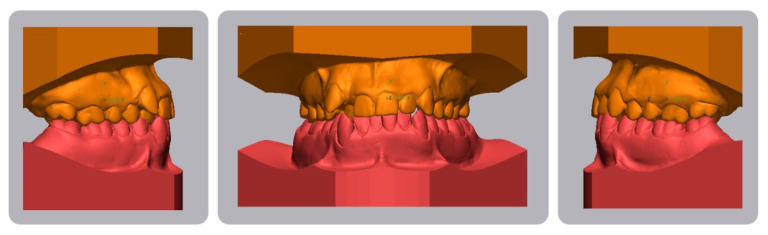
Pretreatment digital casts.

**Figure 4:**
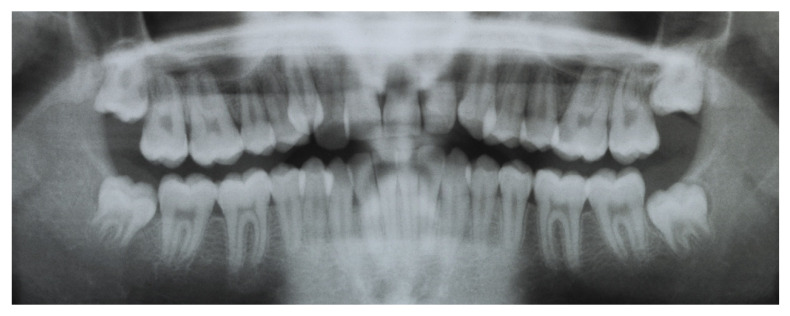
Pretreatment orthopantomogram.

**Figure 5:**
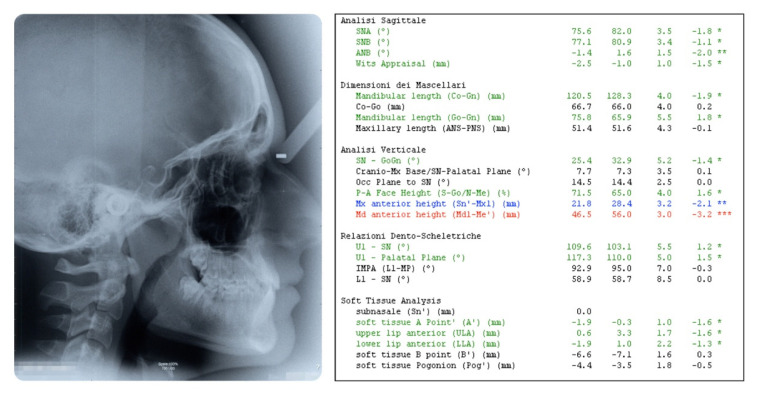
Pretreatment cephalometric analysis. (Value, Norm, Standard Deviation, Deviation Norm).

A 16-year-old male attended a clinical consultation, seeking orthodontic treatment with a chief complaint about his smile aesthetics. The patient presented a flat profile with a moderate skeletal Class III tendency, reduced facial lower height and an acceptable soft tissue projection (Figure 1). The face was relatively symmetric in the frontal view, however, a slight asymmetry of the upper midline relative to the nasal filtrum was evident while smiling. The intraoral examination, dental casts and orthopantomogram revealed a Class I dental relationship with significant crowding in both the maxillary and mandibular arches, with a cross-bite of 1.2 and 2.6, and 1.3 in the buccal ectopic position, and all four third molars’ germs were present (Figure 2, Figure 3 and Figure 4). The maxillary midline was 2 mm off to the right side relative to the facial and mandibular midlines, due to the lateral shift of the central and lateral incisors as a consequence of the ectopic vestibular eruption of the tooth 1.3. The cephalometric analysis confirmed the skeletal Class III tendency in the hypodivergent subject (Figure 5). No temporomandibular disorder (TMD) signs or symptoms were reported or clinically evident. 

### 2.2. Treatment Objectives

The treatment objectives were the correction of both the maxillary and mandibular crowding, placing the maxillary right canine in the Class I position, correcting the cross-bite of the dental elements 1.2 and 2.6 and restore both the maxillary and mandibular midlines; moreover, achieving those occlusal outcomes by increasing the aesthetics of the smile and without worsening the facial profile.

### 2.3. Treatment Alternatives

The first treatment option was to extract both the maxillary and mandibular first premolars. This approach would have allowed for the correcting of both the maxillary and mandibular crowding without increasing the length and width of both dental arches, however, this option would have probably caused a worsening of his facial aesthetics since the patient was bi-retruded with a flat facial profile and thin upper and lower lips. Furthermore, the patients’ parents were opposed to the extraction.

The second alternative was to correct the dentoalveolar discrepancy, achieving a dentoalveolar expansion of both the maxillary and mandibular arches by using a self-ligating fixed appliance [15,16,17,18,19]. This option would lead to better facial aesthetics without the drawback of an increased risk of periodontal tissue alterations. 

Both the orthodontists and the patients’ parents preferred the second option: from the parents’ point of view, it was the more conservative option, from the orthodontists’ point of view, it was the option with the best aesthetic outcomes and in our opinion, the risk of periodontal tissue alterations was mitigated by the increased thickness of the mandibular symphysis that allowed us to correct the mandibular arch crowding through the proinclination of the lower incisors that were initially retroinclinated. 

### 2.4. Treatment Progress

A pre-torqued 0.022 self-ligating fixed appliance with a Damon prescription (Ormco) was bonded to the buccal surface of all the erupted teeth. The same archwire sequence was set for both the maxillary and mandibular arches: 0.014 Thermal NiTi; 0.014 × 0.025 NiTi; 0.017 × 0.025 SS; 0.019 × 0.025 SS. The use of this archwire sequence allowed us to obtain the alignment, leveling and coordination of the maxillary and mandibular arches. The use of thermal archwires during the initial stage of the treatment allowed us to move the teeth through the expression of low force, achieving the correction of the dentoalveolar discrepancy without iatrogenic injuries [20,21,22].

During each appointment, low-level laser therapy (LLLT) was administered using a Diode laser emitting infra-red radiation at 980 nm (Wiser, Doctor Smile—Lambda Spa, Brendola, VI, Italy) in order to accelerate the orthodontic movement, in particular during the alignment stage [23], reduce the patient’s pain and discomfort [24] and increase the metabolic activities in the dentoalveolar complex [25,26]. The total energy density administered in each dental arch was 150 J/cm^2^ according to the previously published guidelines specific for an orthodontic treatment [27]. After 23 months of fixed therapy, the brackets were removed, maxillary and mandibular lingual fixed retainers were placed and Hawley retainers were given to the patient for both the maxillary and mandibular arches.

### 2.5. Results

**Figure 6:**
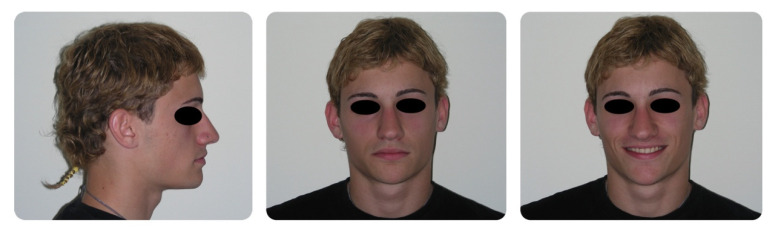
Post-treatment extraoral photographs.

**Figure 7:**
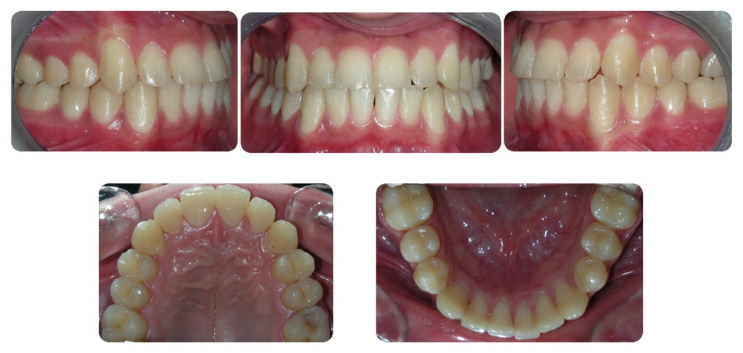
Post-treatment intraoral photographs.

**Figure 8:**
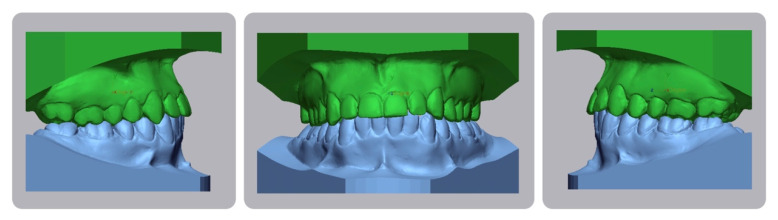
Post-treatment digital casts.

**Figure 9:**
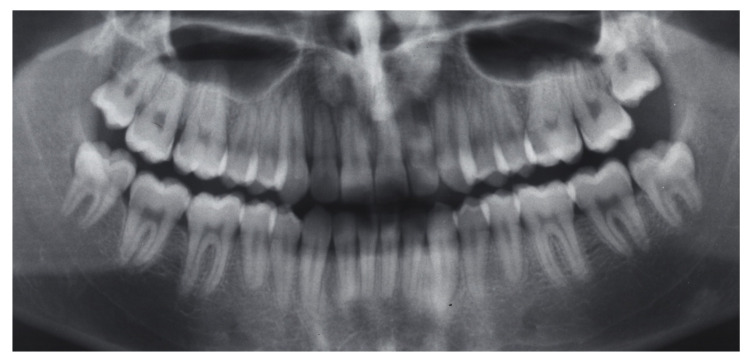
Post-treatment orthopantomogram.

**Figure 10:**
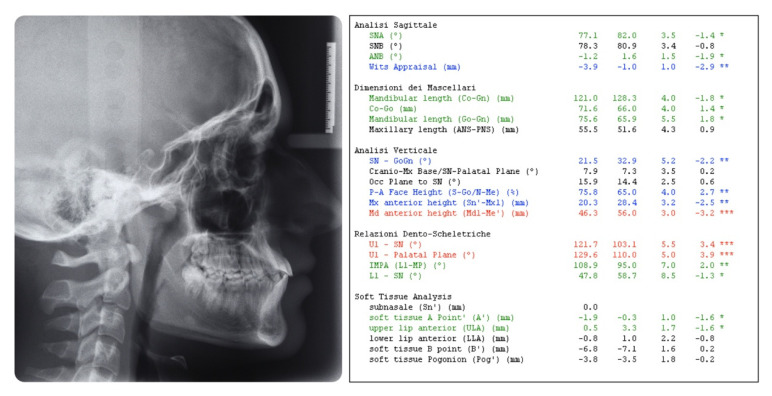
Post-treatment cephalometric analysis. (Value, Norm, Standard Deviation, Deviation Norm).

**Figure 11:**
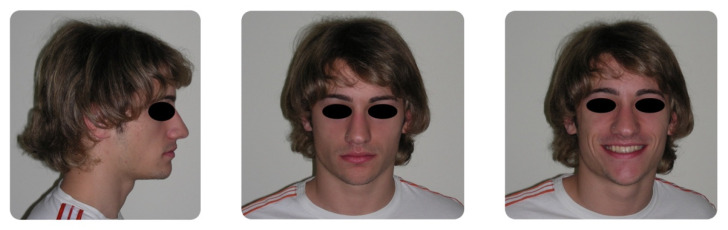
Five-years follow-up, extraoral photographs.

**Figure 12:**
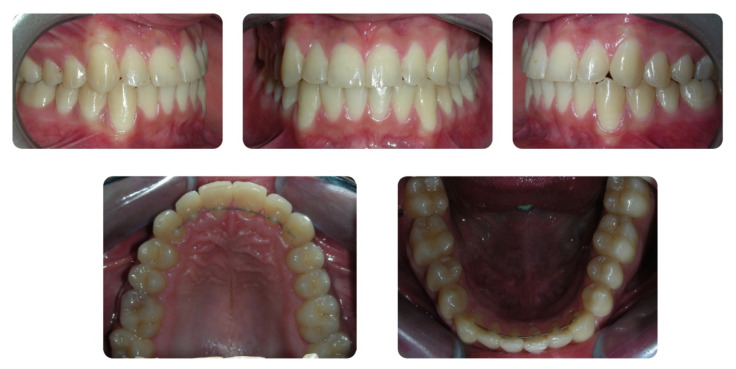
Five-years follow-up, intraoral photographs.

The post-treatment facial and intraoral photographs and dental casts illustrate the significant improvement in the patient’s smile (Figure 6, Figure 7 and Figure 8). The posttreatment orthopantomogram showed the achievement of good root parallelism in the absence of root resorption (Figure 9). The post-treatment cephalometric analysis illustrates the changes achieved with the treatment (Figure 10). The post-treatment dental casts and intraoral photos show a Class I canine occlusion with normal overjet, overbite, canine and incisal guidance. The correction of the crowding has been achieved by the proinclination of both the upper and lower incisors. Furthermore, the proinclination of the upper incisors allowed to camouflage the tendency to Class III thanks to the increase in the dental support of the upper lip. The choice of a non-extraction therapy despite the considerable dentoalveolar discrepancy allowed us to correct the dento-basal discrepancy without a profile modification. Labial competence was maintained despite the considerable proinclination of the upper and lower incisors that occurred as a consequence of the expansive treatment. Looking at the post-treatment lateral cephalogram, it can be seen that the orthodontic treatment did not affect the skeletal facial pattern in this patient. The tendency to Class III has remained unchanged as well as the tendency to a mandibular ante-rotation. The facial and intraoral photographs and dental casts after five years of retention show the results to be stable, both from the occlusal and periodontal point of view (Figure 11 and Figure 12).

## 3. Discussion

This case report shows a borderline patient treated without extraction. By “borderline patient”, we refer to the specific complexity of the decision-making process for planning the orthodontic treatment, considering that both extractive and non-extractive treatment plans would have been two validated options. The will to preserve and improve the patient’s profile as much as possible led us to treat the patient without extraction. The correction of the crowding has been achieved by the proinclination of both the upper and lower incisors, and this outcome could be considered unstable according to some orthodontic schools, such as Tweed’s philosophy, but much evidence reports no differences between an extraction and a non-extraction orthodontic treatment in terms of stability [28,29]. Considering the short- and medium-term results, the decision taken led to satisfying facial aesthetics and a stable occlusion. The current literature reports some evidence for the absence of significant differences between the non-extraction and extraction orthodontic treatments’ respective effectiveness in borderline patients [30,31]. On the other hand, controversial differences in terms of aesthetic outcomes have been summarized, and different studies report a worsening of the facial aesthetics with a more retrusive profile in those patients treated with extraction rather than those treated without extraction, with a significant modification of the lips’ thickness and position, with an increase in the nasolabial angle [30,32]. However, other authors deny this consideration, asserting that the simple statement that extraction means a more retrusive or dished-in profile seems to be unacceptable [33,34]. Many studies report a positive correlation between the buccal corridors and facial aesthetics, and some authors considered extraction treatment to be related to an increase in the buccal corridors due to the narrowing of the arch [35,36,37]. Nevertheless, other evidence has refuted this observation, reporting that there are not significant differences in terms of the arch width and buccal corridors between the extraction and non-extraction treatments [38,39]. Therefore, in regards to many of the most relevant variables related to the decision to extract or not to extract, the current literature does not provide a unanimous opinion. 

In this case report, many secondary variables were considered in the decisional process that led us to treat the patient without extraction: the most relevant was the thickness of the mandibular symphysis. Considering that one of the most important measurements when the orthodontist decides to extract in Class I cases is lower crowding, the possibility or not to correct the lower crowding through the proclination of the lower incisors acquires a decisive role in borderline patients [40,41]. The increase in the mandibular symphysis’ thickness is frequently observed in brachyfacial subjects [42,43,44,45]. Those subjects are usually characterized by an increased masseter muscle volume and consequently with an increased muscular and periodontal activity, which can be related to a greater risk of relapse [45,46,47,48,49,50,51,52,53,54,55,56,57,58].

However, stability cannot be considered as a primary outcome of orthodontic treatment, since a lot of evidence shows that retention protocols are the only way to avoid relapse [50,59,60,61,62,63,64,65,66,67,68,69]. Consequently, the choice to extract or not can be driven by other factors, such as facial type and the mandibular symphysis thickness [70,71,72,73,74,75,76,77,78,79,80,81,82,83,84,85,86]. These parameters nowadays have not been taken too much into account from the literature, however, in our opinion, those factors have great relevance in this complex and insidious decisional process. 

## 4. Conclusions

The current literature does not provide a unanimous opinion in regards to many of the most relevant variables related to the decision to extract or not to extract, so it is not possible to develop an effective decision-making algorithm. Consequently, the orthodontist driven by his own experience and skill should also be able to evaluate many secondary factors, like those highlighted in this paper, in order to make the correct decision.
